# Development of Thin Carbon-Ceramic Based Coatings in Roll-to-Roll Mode: Tribological and Corrosion Results on Stainless Steel

**DOI:** 10.3390/ma18092159

**Published:** 2025-05-07

**Authors:** Mª Fe Menéndez Suárez, Pascal Sanchez, Ana L. Martínez Díez, Beatriz Mingo Roman, Marta Mohedano Sánchez

**Affiliations:** 1 Surface Unit, Fundación Idonial, C/Calafates 11 Avilés, 33490 Asturias, Spain; pascal.sanchez@idonial.com (P.S.); ana.martinez@idonial.com (A.L.M.D.); 2 Departamento de Ciencia de los Materiales e Ingeniería Metalúrgica, Universidad Complutense de Madrid, 28040 Madrid, Spain; beatriz.mingo@manchester.ac.uk (B.M.R.); mmohedano@quim.ucm.es (M.M.S.)

**Keywords:** coatings, sol-gel, carbon nanomaterials, corrosion, tribology, graphene nanoplatelets

## Abstract

In this work, silicon oxide based coatings with embedded graphene nanoplatelets (content ranging from 1.8 wt.% to 7.2 wt.%) have been developed following the sol-gel route, using AISI430 stainless steel as substrate and dip and roll-to-roll as coating techniques. The tribological and corrosion behaviour of these coatings have been evaluated and compared to bare steel. Concerning tribological behaviour, the coefficient of friction and wear print were significantly reduced with increasing the graphene nanoplatelets content. Regarding corrosion, all coatings showed improved corrosion behaviour compared to bare steel. However, higher concentration of nanoplatelets revealed a negative effect on the corrosion resistance, probably due to aggregation. Taking into account these two counteracting effects, as final part of this work, a bilayer coating with different graphene content has been proposed and fabricated. A top layer, with high graphene nanoplatelets concentration has allowed enhanced tribological properties whereas bottom layer, with no graphene nanoplatelets assures corrosion inhibition under harsh environments.

## 1. Introduction

In the last decade, graphene and graphene oxide materials have been deeply studied because of their demonstrated anti-corrosive and anti-oxidative properties in different metallic substrates, as copper or nickel (see, for example [1,2]). In general, these initial experiments have been carried out by means of synthesis and/or transferring of chemical vapour deposition (CVD) grown graphene because of their high quality (in terms of control of the number of layers, coverage and lack of imperfections) in spite of evident technical and economic difficulties for industrial implementation of these coatings. Although the use of exfoliated graphene and graphene oxide platelets for corrosion protection was initially applied to copper electrical contacts [3], a great interest has emerged in steel industry in the last years leading to graphene based coating with protective functionality. Main results have been achieved by the use of coating techniques as spin coating [4] or stacking graphene nanoplatelets in multilayered protective layers deposited by physical vapour deposition (PVD) [5]. In general, the good performance of these materials is mainly due to their layered structure that represents a drastic increment in diffusion length for corrosive elements.

In parallel, the use of graphitic materials as solid lubricants is well known since decades [6,7]. Microsized graphite powder has been embedded in different matrixes in order to provide good tribological performance (see, for example [8]). The size of these particles has mainly driven to thick polymeric coatings (a few hundred microns). In recent years, tribological properties of graphene and graphene oxide nanocoatings have also paid great attention due to intrinsic toughness and low shear strength [9]. Reza et al. [10] have proposed the use of water as lubricant replacing oil when reduced graphene oxide materials are deposited. However, the use of single layer graphene grown by CVD or few-layered exfoliated graphene oxide (latter reduced) presents nowadays important drawbacks facing industrial implementation, mainly because of economic viability.

Concerning base material of the coating (matrix), sol-gel technology has represented a good alternative to polymeric coatings on steel since 1992 [11] with renewed interest in the last decade [12,13]. This process allows the synthesis of high dense ceramic coatings (e.g., SiO_2_, Al_2_O_3_, ZrO_2_) in a simple, cheap, low temperature and non-vacuum way. The densification of the films presents problems when thicknesses above a few microns are required, what limits the embedment of microsized particles. However, sol-gel matrixes represent a perfect matrix for nanosized particles; and, particularly for carbon nanomaterials, several researches have been reported in the last years. Parhizkar et al. [14] investigated the effect of graphene oxide nano-fillers embedded in silicon sol-gel matrix on the corrosion protection and adhesion properties of an epoxy coating on a steel substrate pre-treated with silane. They observed a clear improvement of the adhesion strength and a reduction of the corrosion rate. Xue et al. [15] carried out sol-gel layer functionalised with graphene oxide on AA2024-T3 aluminium alloy. They reported that a concentration of 0.5 mg/mL of graphene oxide improved the corrosion protection (corrosion current density one order magnitude smaller than the sol-gel film without graphene oxide) due to an enhancement of the cross linking of the layer though a covalently reaction of the graphene oxide with the silanol groups. Li et al. [16] developed a composite film combining sol-gel layer filled with graphene oxide and a plasma electrolytic oxidation layer on titanium alloy. They studied the tribocorrosion properties of this composite and reported that the addition of graphene oxide improved both the corrosion performance and the wear resistance due to the formation a good barrier and lubricant graphene oxide nanosheets.

Taking into account the aforementioned facts, this work reports the tribological, wear and corrosion properties of silicon oxide (SiO_x_ with x ≅ 2) sol-gel coatings using graphene nanoplatelets (GP) as an additive and considering a stainless steel substrate (AISI430). In this way, oblate GP with thickness of 7 nm have been considered as a good compromise between conventional graphite micropowder and few-layered reduced graphene oxide (RGO), in terms of technical performance and cost. Graphene nanoplatelets have been embedded in a SiO_x_ matrix developed by sol-gel processes in order to provide a robust inorganic structure with strong adherence and flexibility on stainless steel. As first step, a single layer coating was selected for simplicity and industrial viability. Obtained results on these initial tests have allowed designing and manufacturing a bilayer coating with doped/undoped structure in order to surpass problems in corrosion performance mainly due to graphene nanoplatelets aggregation.

## 2. Experimental

### 2.1. Materials

**Figure 1:**
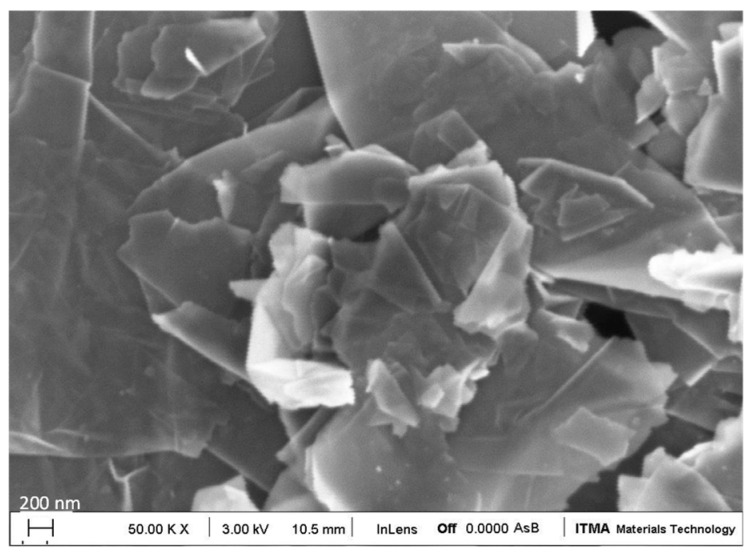
FEG-SEM image of graphene platelets.

AISI430 (Alloy430) is the substrate used in this work because is one of the most used ferritic stainless steel grade in the world. A medium chromium content of 16 wt.% gives better corrosion resistance and this grade is used in a wide range of applications because of its superb compromise between cost and technical performance. Table 1 shows the chemical composition of this selected steel. Graphene nanoplatelets were purchase from Graphene Supermarket in ultra-high concentrated dispersion (23 wt.%), average thickness of 7 nm and diluted in n-butyl acetate (Figure 1).

### 2.2. Coatings

The sol formulation followed a one-step acid catalysis using Methyltriethoxysilane (MTES 99%, Aldrich, Darmstad, Germany) and Tetraethylorthosilicate (TEOS 98%, Aldrich, Darmstad, Germany) as precursors. Water was incorporated as a solvent, polyethylene glycol (PEG-6000, Panreac, Barcelona, Spain) as tension releasing agents and nitric acid (HNO_3_) as a catalyst. The optimized molar ratios used in order to achieve good homogeneity, adhesion and absence of cracks after curing were TEOS/MTES: 1.35, H_2_O/(MTES+TEOS): 4.7, and PEG-6000/(MTES+TEOS): 0.02. In addition, polyvinyl pyrrolidone (PVP10000, 99%, Aldrich, Darmstad, Germany) was also added as a tension releasing agent and also to increase the solution viscosity in order to get a better control of thickness. The optimized molar ratio used was PVP/(MTES+TEOS): 0.02.

The synthesis was carried out at room temperature under vigorous stirring. The addition of HNO_3_ sets off the hydrolysis, increasing the temperature by 25 °C. During the sol phase preparation, the ambient conditions have been controlled, being the average temperature registered of 23 °C and the relative humidity of 48%.

The sol-gel surface tension was also studied in order to assess the suitability of this SiO_x_ based coating with the steel substrate in terms of wetting and adhesion capability as well as its compatibility with printing and coating techniques. A Dynamic Absorption and Contact Angle Tester FIBRO DAT 1100 was used for surface tension measurements taking into account the pendant drop method. The sol-gel was pumped through a needle and optimized drops of (8.0 ± 0.1) μL were used. The drops (five replica) were then optically observed and the surface tension was calculated from the shape of the drop using software that is part of the equipment. The surface tension was (26.19 ± 0.14) mN/m, which seems to be compatible with all printing and coating technologies.

The solution was deposited by means of dip-coating technique at lab and pilot plant scale (roll-to-roll; SmartCoater, Coatema GmbH, Dormagen, Germany). In the first case, 50 × 50 mm^2^ samples were considered. For pilot plant tests, a coil 50 mm wide and 1000 mm long was coated in these preliminary tests.

As final step, the films were thermally cured following a low temperature treatment. In this way, the sample was initially held for 30 min at 60 °C, then 150 °C for 15 min, and finally 210 °C for 60 min.

### 2.3. Characterization

A complete characterization of the coating morphology was conducted by confocal microscopy (Olympus LEXT OLS300, Tokyo, Japan), field emission electron microscopy (Carl Zeiss SMT Ultra Series, Oberkochen, Germany) and Raman spectroscopy (Jobin Ybon Horiba HR800, Longjumeau, France).

### 2.4. Tribological Tests

The tribological properties of the coatings were tested in dry conditions at room temperature (23 °C) using a ball on disc tribotester (Bruker, UMT3, Karlsruhe, Germany) following the ASTM G99 standard. The coating served as discs and AISI 52100 balls of 9.5 mm in diameter were used as counterpart. The tests were carried out during 5 min, at 5 N normal load, 15 Hz of frequency and 4 mm of track distance. The wear resistance was evaluated monitoring the friction coefficient and the wear rate was calculated from the worn volume obtained by confocal/interferometric microscopy (Leica DCM 3D, Manheim, Germany). Five replica were made for each sample.

### 2.5. Corrosion Tests

Polarization curves were obtained after one hour of immersion in naturally-aerated 3.5 wt.% sodium chloride solution at room temperature (22 °C) using a Gill AC potentiostat connected to a three-electrode cell. The working electrode was the test material with an immersed area of 1 cm^2^ and graphite and silver/silver chloride (Ag/AgCl) electrodes were used as the counter and reference electrodes respectively. Solution concentration inside the reference electrode compartment was 3 M KCl, providing a potential of 0.197 V with respect to the standard. Polarization curves were measured at a scan rate of 10 mV/min, from −200 mV with respect to the open circuit potential (OCP) until a maximum current of 5 mA/cm^2^. From the polarization curves, corrosion and breakdown potentials (E_corr_, E_br_, respectively) and corrosion current density (i_corr_) values were determined. Measurements were performed twice to ensure reproducibility of the results.

## 3. Results

### 3.1. Morphology and Thickness

**Figure 2:**
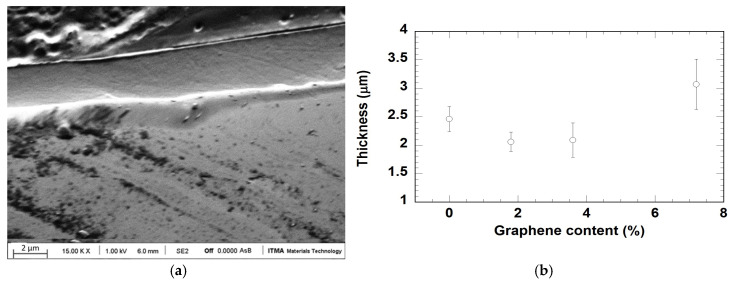
(**a**) FEG-SEM image corresponding to 3.6 wt.% graphene SiO_x_ coating on AISI430. (**b**) Measured thickness of the developed coatings as function of graphene nanoplatelets content.

Figure 2a shows the scanning electron microscopy image of one of the coatings developed in this work (3.6 wt.% of graphene content). Similar results were obtained for all the coatings. In all cases, the cross section shows dense and homogeneous layers. Concerning thickness, an average value of 2.42 μm, with no clear differences depending on graphene content, under experimental uncertainty, as it can be seen in Figure 2b.

**Figure 3:**
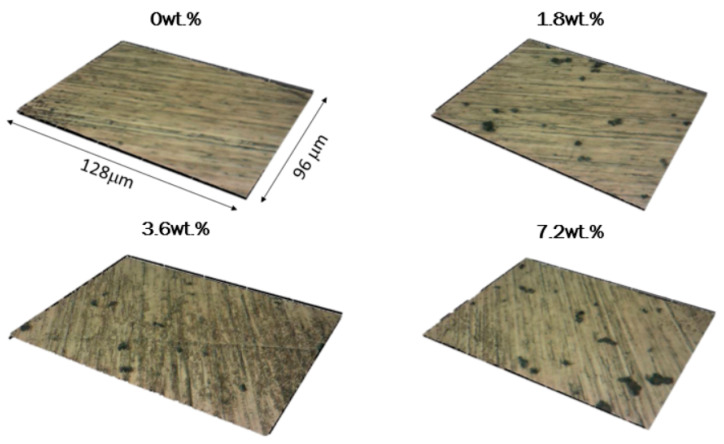
Confocal topography (128 μm × 96 μm) of the coatings. Real microscopy image caption has been superimposed for clarity.

Figure 3 represents the confocal topography of coated steel samples, with real image superimposed.

**Figure 4:**
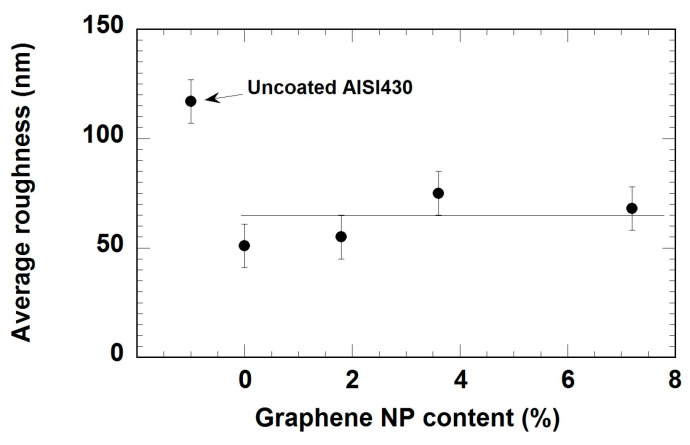
Average roughness of the coatings as function of graphene nanoplatelets content.

In all cases, the average roughness (Ra) of all the samples was evaluated from these measurements and represented in Figure 4. It can be observed that the sol-gel coatings reduce the average roughness of bare steel. The absence of an increase of roughness with graphene content seems to indicate the low aggregation of graphene nanoplatelets in the surface of these coatings.

**Figure 5:**
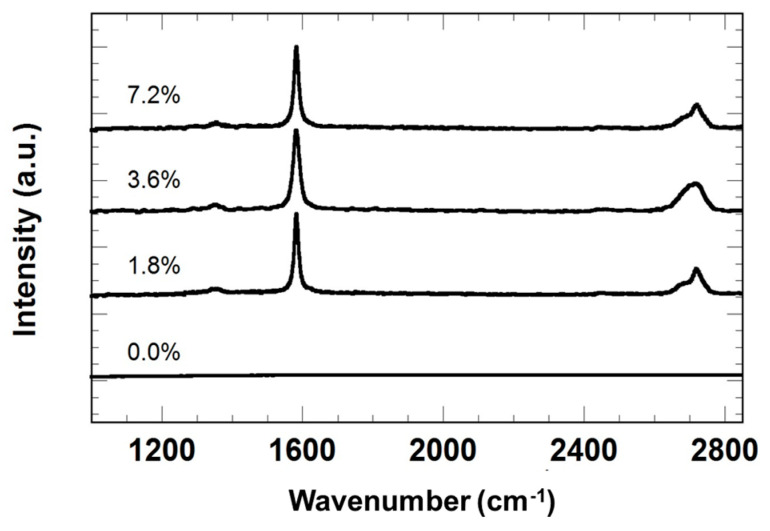
Raman spectra of the coatings as function of graphene nanoplatelets content. The curves have been vertically displaced for clarity.

The Raman spectrum of the graphene nanoplatelets in the sol-gel coating are represented in Figure 5, showing characteristic D, G and 2D bands at 1352 cm^−1^, 1582 cm^−1^ and 2719 cm^−1^, respectively. The ratio of the D to G band intensity is 0.09 in all the cases. The lack of symmetry in the 2D band is associated to the perfect stacking of the graphene layers in the nanoplatelets [17], what makes these spectra to be more similar to graphite than to few-layered graphene.

### 3.2. Tribology and Wear

**Figure 6:**
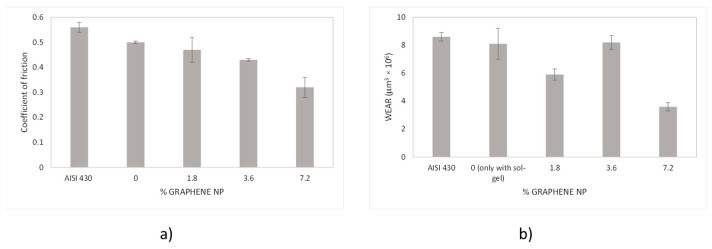
(**a**) Average values of coefficient of friction and (**b**) volume loss values for all the coatings as function of graphene nanoplatelets content.

The coefficient of friction was measured for all the developed coatings as a function of time for the 300 s time length. Figure 6a shows the average value for this experiment. A clear reduction of this parameter as function of graphene content is observed under experimental uncertainty, being this decrease more noticeable for 3.6 wt.% and 7.2 wt.% samples. This indicates the enhancement of tribological behaviour above a threshold, as observed by other authors using graphene nanostructures as lubricant additives [18]. Graphene-based nanostructures are extremely hard and provide high mechanical and lubricant properties. The introduction of the graphene platelets in the SiO_x_ sol-gel matrix, in addition to avoid a direct metal-metal contact, possibly forms a tribofilm with higher hardness than the sol-gel alone and enable the decrease of both friction coefficient and wear resistance. The increase of graphene-platelets contains gives a higher hardness to the coating and lower friction coefficient values.

Wear resistance measurements show no so direct relationship with graphene content as in the case of tribological tests. However, a clear a reduction of volume loss values is observed for 7.2 wt.% sample, as shown in Figure 6b. The high wear value for the 3.6 wt.% condition may be explained by an heterogeneous dispersion of the graphene platelets not embedded in the SiO_x_ sol-gel matrix and its higher concentration at the surface. As consequence, during the ball on disc trial, a high amount of graphene platelets is easily removed with a low friction level together with sol-gel film that directly support the graphene platelets.

### 3.3. Corrosion

**Figure 7:**
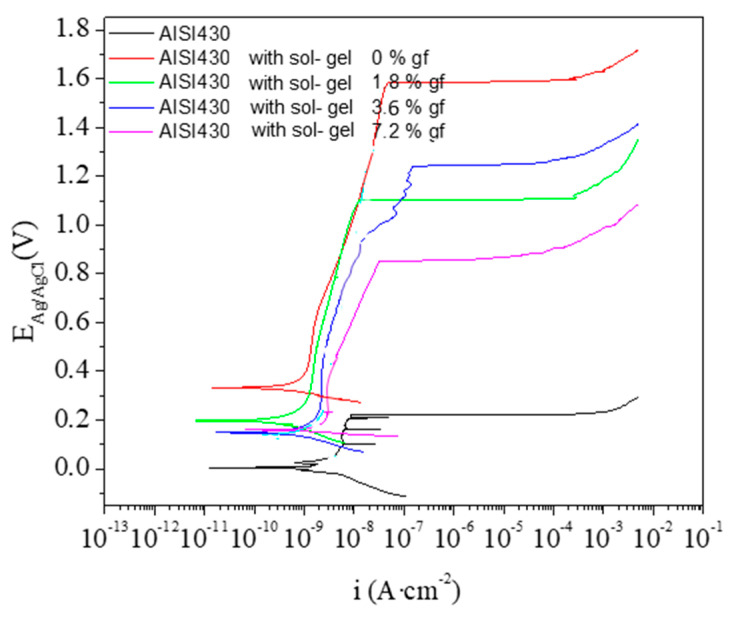
Potentiometric curves of coatings as function of graphene nanoplatelets content.

Figure 7 shows the potentiodynamic polarization curves after 1 h of immersion in naturally-aerated 3.5 wt.% NaCl solution at room temperature for all the studied materials. The corresponding parameters derived from the potentiodynamic curves are listed in Table 2.

The uncoated material shows a E_corr_~4 mV and a passive region (E_bd_-E_corr_) of ~210 mV, delimitated by the E_bd_, which is manly formed by Cr_2_O_3_. All coated materials show better corrosion resitance compared to the substrate as deduced from the displacement of the E_corr_ to more noble potentials and to the shift of the current densities to lower values. Additionally, the passivity is also improved as it can be observed in the greater (E_bd_-E_corr_) values in Table 2. However, the incorporation of nanoplatelets to the coating seem to have a slighly negative influence in the corrosion resistance, since the polarization curves shift toward higher current densities and the passivity regions decreases as the concentration of nanoplatelets increases, suggesting the formation of slightly worse protective layers. Probably, the presence of nanoplatelets partially disturbs the continuity of the coatings, acting as weak points promoting corrosion initiation.

## 4. Discussion

From tribological tests, it can be said that adding graphene nanoplatelets not only improves the performance of the coating in terms of reduction of the coefficient of friction but also contributes to minor wear volume, where the COF has been greatly reduced in a 40%, and the wear rate in a 50% respect the sol-gel without graphene nanoplatelets, especially in the sample with higher graphene content (7.2 wt.%).

**Figure 8:**
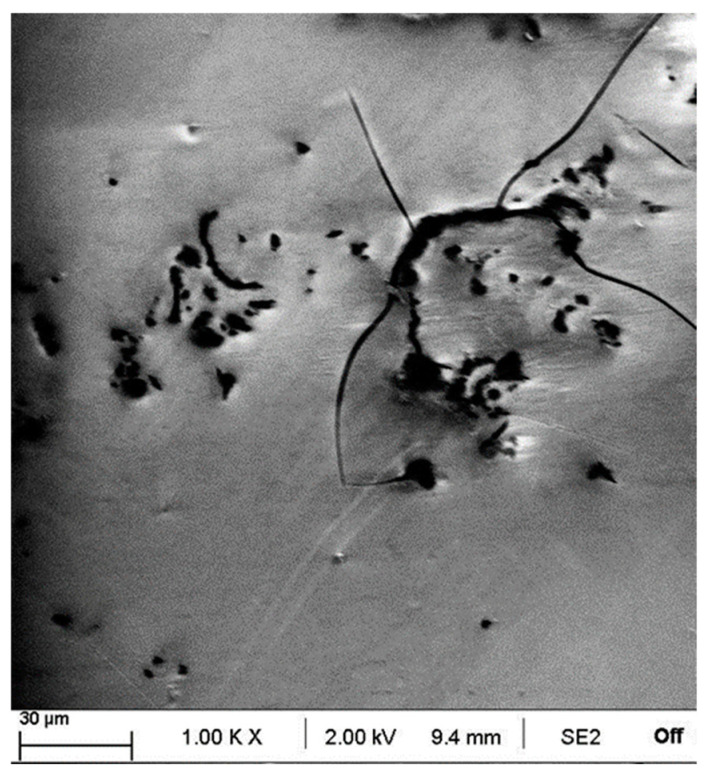
Graphene nanoplatelets agglomeration in sol-gel coating surface for the 7.2 wt.% sample.

Potentiometric analysis show the corrosion inhibiting properties of the developed coatings under harsh conditions when compared to bare steel. However, graphene content seems to have a negative effect on this behaviour. The more plausible reason is that partial agglomeration of graphene nanoplatelets in a little extent induce cracking and consequent base material exposition to the corrosive medium. As some authors have modelled, the presence of small cracks in a low proportion of total area can drive to large corrosion because of underetching [19]. In this sense, it has been recently studied the effect of graphene nanoplatelets content in polymer matrixes showing that fracture toughness is enhanced up to a limit value; beyond it, the addition of graphene nanoplatelets has a negative effect down to non-doped matrix [20]. In this work, some of these defects have been found under microscopic analysis with more noticeable presence with increasing graphene nanoplatelets content. Figure 8 shows a dramatic failure in the 7.2 wt.% sample with visual crack propagation.

**Figure 9:**
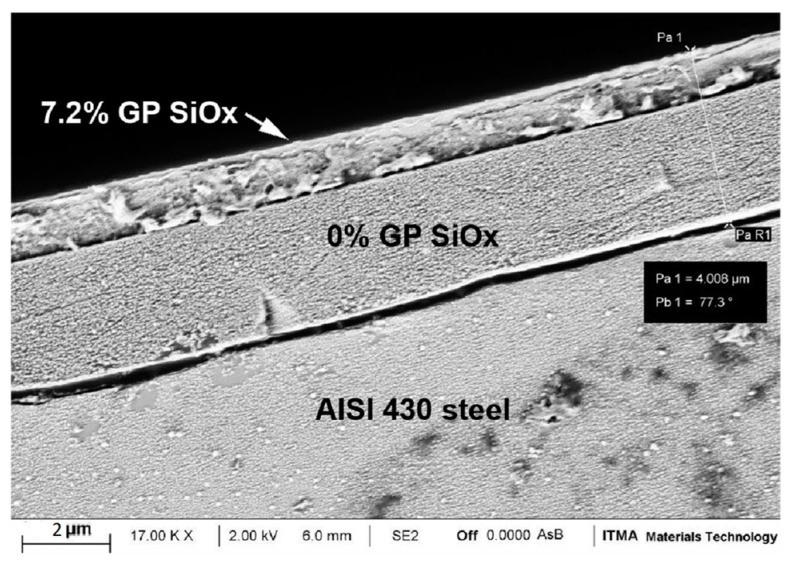
FEG-SEM image corresponding to the manufactured bilayer coating.

Taking into account the counteracting effect of graphene nanoplatelets on tribology/wear and corrosion properties, a bi-layer structure has been designed and developed in order to fulfil both functionalities. In this way, the upper layer of this structure, with a high content of graphene nanoplatelets (7.2 wt.%) will enhance tribological and wear properties to the coating. Underneath, a sol-gel layer with no graphene content will protect the stainless steel surface from corrosion. Figure 9 presents the manufactured bi-layer coating under the same experimental procedure followed for the previously developed single layer coatings.

**Figure 10:**
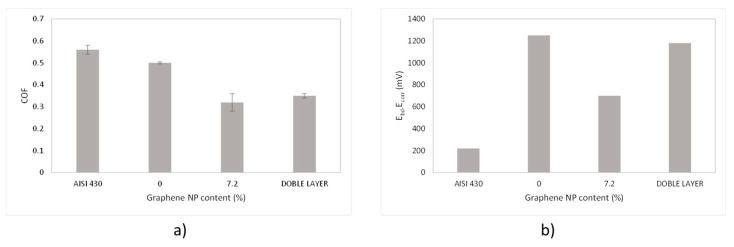
(**a**) COF average values for all the studies samples, including bare steel and bilayer. (**b**) Passivation region for all the samples as extracted from potentiometric curves.

Figure 10a shows the coefficient of friction of this bilayer system compared to single layers and bare steel. As expected, this value is close to the 7.2 wt.% sample under experimental uncertainty. Figure 10b presents the pasive region (E_bd_-E_corr_) of all the samples, as extracted from Figure 7 and listed in Table 2. In this case, it is also clear that the behaviour of this new bilayer concept is similar to 0 wt.% sample, as it was desired.

## 5. Conclusions

SiO_x_ inorganic coatings with embedded graphene nanoplatelets have been developed by sol-gel at lab and pilot plant scale using stainless steel as substrate. These coatings seem to improve the performance of the substrate in terms of the reduction of friction and corrosion. In the first case, it can be concluded that the addition of graphene nanoplatelets drives to a reduction of the coefficient of friction above a threshold concentration. In the case of corrosion, coatings with low graphene concentration show better performance, probably due to small aggregation of nanoplatelets and subsequent cracking of the film and under etching.

As a final part of this work, a bilayer coating has been developed comprising non-graphene doping in the inner side acting as corrosion inhibitor and an outer graphene-doped layer to enhance the tribological performance of the coating. In this way, this new coating possesses enhanced tribological properties and corrosion inhibition, what constitutes the most innovative part of this paper.

## Figures and Tables

**Table 1:** Chemical composition of the stainless steel used as substrate in this work.

	Chemical Composition % (wt.)					
	C	Si	Mn	P	S	Cr
AISI 430	≤0.08	≤1.0	≤1.0	≤0.04	≤0.03	16.0–18.0

**Table 2:** Corrosion parameters calculated from potentiodynamic measurements for bare and coated steels.

Materials	E_corr_ (mV)	E_bd_ (mV)	E_bd_-E_corr_ (mV)	I_corr_ (mA/cm^2^)
AISI430 (uncoated)	4	220	216	5.077 × 10^−7^
AISI430 + 0 wt.% GP	334	1571	1237	1.198 × 10^−7^
AISI430 + 1.8 wt.% GP	201	1104	903	3.122 × 10^−7^
AISI430 + 3.6 wt.% GP	151	1235	1084	3.057 × 10^−7^
AISI430 + 7.2 wt.% GP	161	841	680	5.990 × 10^−7^

## Data Availability

All the data presented in this study are contained within the article.
